# Australian Notes

**Published:** 1918-08-31

**Authors:** 


### AUSTRALIAN NOTES.

(FROM OUR AUSTRALIAN CORRESPONDENT.)

### Hospital Association for Australia.

On May 15 delegates from some forty country hospitals met at Melbourne Town Hall and agreed to form an Association, to be named the Country Hospitals Association of Victoria, to promote their financial and other interests, including those relating to Government subsidies and municipal assistance. Mr. R. J. Fincham (Horsham), convener of the meeting, was elected president, Messrs. Thomas Graham (Wangaratta) and S. I. McGowan (Bendigo) vice-presidents, and Mr. J. Jessop (Amherst) hon. treasurer.

Possibly fired by this example, Mr. McPherson a few days later favoured the formation of an Association of city hospitals, with a board, to decide how best the £60,000, or whatever the Government had to give, might be allotted. He could not say that the Ministry would bring in a Bill this year, but a board, he felt, was certainly wanted. The Board of Health has just condemned a wing of the Women's Hospital, which will cost £30,000 to rebuild, and the Children's Hospital wishes to erect a new wing, at an estimated cost of £11,000, to accommodate babies up to two years. But the State Treasurer, in iterating his belief in the efficiency of the board mentioned, has said nay to both in view of the present cost of building. Any hospital that can show that it has suffered financially through the cutting down of the grant is promised re-imbursement.

### The Working of the Venereal Diseases Act.

Despite the regulations of the Venereal Diseases Act passed last year, in the State of Victoria matters remain unsatisfactory, especially for women sufferers. There is no clinic open to them except at general hospitals. The Queen Victoria Hospital has come to the rescue, and is to co-operate. On vacant ground next the Queen Victoria Hospital the Government is about to build temporary quarters as a clinic for women, who will be attended by the medical staff of the Queen Victoria Hospital pending the erection of a completely equipped Lock Hospital and an arrangement with existing hospitals. The Alfred Hospital has opened its clinic, and as many as sixty cases a night are being treated. The Children's Hospital has agreed to treat infants, and is to receive from Government £600 per annum for the maintenance of ten beds. In the ten months July 1, 1917, to April 30, 1918, 6,346 cases have been reported, among them a few doctors and nurses who had contracted the disease while attending midwifery cases. Latest information is that plans are being prepared for the £5,000 hospital.

### A Hospital Clearing-House.

Sydney's Hospital Admission Depôt has been found extremely useful in saving time for patients and accelerating their treatment. Persons calling there are examined and then sent off to the hospital proper for their complaint, whereas in Melbourne sick people have sometimes been unable to find asylum because the hospital is shy of admitting unusual cases, and the ailment may not be one treated at the hospital to which they apply. The patients themselves often do not know to which hospital to go. Melbourne may found a similar institution.

### Women Pharmacists.

In the Senate (Federal) the Minister for Defence on May 23 announced that arrangements were being made to employ female pharmacists in military hospitals. Later, in Sydney, Judge Curlewis delivered his award for pharmacists, graded as follows :—(*a*) Manager, £4 10s. a week of forty-eight hours, and time and a quarter thereafter. (*b*) Chief assistant, £4 2s. 6d. (*c*) Registered assistant, £3 16s. 6d. (*d*) Unregistered assistant, six years' experience, £3 7s. 6d. (*e*) Apprentice assistant, five years' experience, £3 3s. ; four years' experience, £2 10s. ; three years' experience, £2 ; two years' experience, £1 7s. 6d. ; one year's experience, £1. (*f*) Reliever, 25 per cent. in excess of the rates for assistants of like grades. No male employee over twenty-one years to receive less than £2 15s. 6d. a week.

### A Patient's Contra Account.

THE *South African Medical Record* tells a good story of a medical practitioner who, when visiting a regular patient, found he had forgotten to bring a lancet, which he required. He, very courteously, asked the father of the patient if he would mind fetching it from his house, which, by the way, was only just across a square. The father went, apparently with the greatest willingness, and fetched the lancet. In due course an account was sent in by the medico. A cheque came promptly, but with five shillings deducted, this *contra* entry in the doctor's account being explained as follows : "To visiting Dr. Blank's house at his request, for the purpose of procuring an instrument, 5s." The doctor at first thought of sending a legal demand for the odd 5s., but, on second thoughts, decided that as this patient—a German, by the way—valued his own time at 5s. a visit, he probably would not object to his doctor valuing his at a higher rate, so he resolved, in all future accounts, to increase his charges per visit to this house from 5s., which had hitherto been the figure, to 7s. 6d. The patient has regularly paid this higher charge without comment, and the parties continue on the best of terms.

### Recreation Hut at Chester War Hospital.

A RECREATION hut has now been opened for the use of the patients at the above hospital by Lady Pitcairn Campbell, who was accompanied on the occasion by Lieut.-General Sir W. Pitcairn Campbell, K.C.B., G.O. Commander-in-Chief Western Command. A large number of guests was present.

Lieut.-Col. Prout, C.M.G., Officer Commanding Chester War Hospital, outlined the steps taken to build the hut, and Mr. Powell, who took charge of the recreation account on the departure of General Sir William Douglas, one of the originators of the scheme, gave some details of what had been accomplished.

After Lady Pitcairn Campbell had declared the hut open, General Sir Pitcairn Campbell presented medals to Sergt. B. A. Flynn, of the Royal Irish Fusiliers, and to Pte. C. H. Buzzard, Lancashire Fusiliers, for gallantry on the field. Both are patients of the Chester War Hospital.

